# Comparative Study of Diethylaminoethyl-Chitosan and Methylglycol-Chitosan as Potential Non-Viral Vectors for Gene Therapy

**DOI:** 10.3390/polym10040442

**Published:** 2018-04-14

**Authors:** Sergei V. Raik, Stanislav Andranovitš, Valentina A. Petrova, Yingying Xu, Jenny Ka-Wing Lam, Gordon A. Morris, Alexandra V. Brodskaia, Luca Casettari, Andreii S. Kritchenkov, Yury A. Skorik

**Affiliations:** 1 Institute of Macromolecular Compounds of the Russian Academy of Sciences, Bolshoi pr. VO 31, St. Petersburg 199004, Russia; raiksv@gmail.com (S.V.R.); valentina_petrova_49@mail.ru (V.A.P.); platinist@mail.ru (A.S.K.); 2 Institute of Chemistry, St. Petersburg State University, Universitetskii pr. 26, Petrodvorets, St. Petersburg 198504, Russia; 3 University of Vienna, Althanstrasse, 14, A-1090 Vienna, Austria; stanislav.andranovich@pharminnotech.com; 4 Department of Biomolecular Sciences, School of Pharmacy, University of Urbino, 61029 Urbino, Italy; luca.casettari@uniurb.it; 5 Department of Pharmacology & Pharmacy, Li Ka Shing Faculty of Medicine, The University of Hong Kong, 21 Sassoon Road, Pokfulam, Hong Kong, China; yingyingxu2010@hotmail.com (Y.X.); jkwlam@hku.hk (J.K.-W.L.); 6 Department of Chemical Sciences, University of Huddersfield, Huddersfield HD1 3DH, UK; g.morris@hud.ac.uk; 7 Research Institute of Influenza, ul. Prof. Popova 15/17, St. Petersburg 197376, Russia; alexandra.b_05@mail.ru; 8 Peter the Great St. Petersburg Polytechnic University, Polytechnicheskaya ul. 29, St. Petersburg 195251, Russia; 9 Institute of Experimental Medicine, Almazov National Medical Research Centre, ul. Akkuratova 2, St. Petersburg 197341, Russia

**Keywords:** diethylaminoethyl-chitosan, methylglycol-chitosan, polyplex, cell transfection, gene delivery

## Abstract

In this paper, we compared the transfection efficiency and cytotoxicity of methylglycol-chitosan (MG-CS) and diethylaminoethyl-chitosan (DEAE-CS_I_ and DEAE-CS_II_ with degrees of substitution of 1.2 and 0.57, respectively) to that of Lipofectamine (used as a reference transfection vector). MG-CS contains quaternary amines to improve DNA binding, whereas the DEAE-CS exhibits pH buffering capability that would ostensibly enhance transfection efficiency by promoting endosomal escape. Gel retardation assays showed that both DEAE-CS and MG-CS bound to DNA at a polysaccharide:DNA mass ratio of 2:1. In Calu-3 cells, the DNA transfection activity was significantly better with MG-CS than with DEAE-CS, and the efficiency improved with increasing polysaccharide:DNA ratios. By contrast, the efficiency of DEAE-CS_I_ and DEAE-CS_II_ was independent of the polysaccharide:DNA ratio. Conversely, in the transfection-recalcitrant JAWSII cells, both Lipofectamine and MG-CS showed significantly lower DNA transfection activity than in Calu-3 cells, whereas the efficiency of DEAE-CS_I_ and DEAE-CS_II_ was similar in both cell lines. The toxicity of DEAE-CS increased with increasing concentrations of the polymer and its degree of substitution, whereas MG-CS demonstrated negligible cytotoxicity, even at the highest concentration studied. Overall, MG-CS proved to be a more efficient and less toxic transfection agent when compared to DEAE-CS.

## 1. Introduction

Gene therapy has attracted substantial attention due to its vast therapeutic potential for the treatment of various diseases, including genetic disorders, cancer, and infections [1]. Another application of gene therapy is the use of DNA as a vaccine to induce immunity against infectious diseases and cancers. However, despite the promising prospects of gene therapy, the lack of highly efficient and safe gene carriers represents a major barrier to its clinical translation [2,3,4,5]. The design and development of effective and nontoxic gene delivery vectors are therefore priorities in gene therapy.

Gene delivery systems can be broadly divided into two categories: recombinant virus vectors and synthetic vectors. Viral vectors have shown good transduction efficiency in in vivo experiments, but their use in clinical practice is severely limited because of their serious side effects (e.g., immunogenicity, carcinogenicity, etc.) and the difficulties associated with vector modification [6]. These limitations have prompted the development of non-viral gene carriers, which typically consist of different synthetic cationic polymers, including poly(ethylene)imine, poly(l-lysine)-*grafted*-imidazole, and poly(β-amino)esters. However, these polymers are also limited in their application due to their pronounced cytotoxicity [5,7].

By contrast, natural polymers, like chitosan, demonstrate transfection efficiency comparable to that achieved with synthetic cationic polymers, but they show low toxicity and good in vivo biosafety profiles [8,9,10]. Chitosan is a popular choice for gene transfer studies because of its low cost, excellent biocompatibility, and notable biodegradability. Moreover, the abundance of primary amines and hydroxyl groups in the chitosan backbone favors tunable chemical modification of the polymer to enhance its efficacy for gene delivery [11,12].

The transfer of an exogenous gene to the cell nucleus requires that a series of extracellular and intracellular obstacles first be overcome. Viruses have evolved functions to address each of these obstacles, but synthetic vectors usually lack the necessary mechanisms. The key design criteria for non-viral vectors must allow for: (i) condensation and protection of DNA; (ii) cellular internalization; (iii) endosomal escape; (iv) transport through the cytoplasm; and (v) nuclear localization [13,14]. Of these steps in the gene delivery process, transport through the cytoplasm and nuclear localization are the most inadequately investigated, and the weak characterization of the underlying mechanisms impedes the design of improved polymeric vectors. By contrast, DNA condensation, cellular internalization and endosomal escape have been relatively well investigated in polymeric gene delivery vectors [5,15].

Gene delivery vectors bind to and condense DNA into small compact structures through electrostatic interactions between the positive charges displayed on the vector backbone and the negative phosphates along the DNA chain. The process of DNA condensation is entropically driven, so a cationic polymer and DNA spontaneously form polyplexes upon mixing [16]. These polyplexes protect DNA by sterically blocking the access of nucleases [17].

Once DNA is bound and condensed, the next obstacle that the delivery vector must overcome is cellular internalization. Efficient cellular uptake requires careful optimization of various parameters that affect cell-surface binding. These can range from the balance between specific targeting interactions and nonspecific electrostatic binding to the cell surface and the narrow window of the polymer:DNA ratio to the optimal ligand valency due to saturation of both receptor binding and the cell’s internalization machinery [18]. Following successful cellular uptake, however, the polyplexes may become entrapped in the endosomes.

One effective strategy for overcoming the endosomal barrier is the use of the proton-sponge polymers that contain a large number of secondary and tertiary amines with p*K*_a_ values that lie in a range between the lysosomal and physiological pH (i.e., pH 4.5–7.2). In the low-pH endosomal environment, the proton-sponge polymers undergo acidification and become protonated, which triggers an influx of more protons into the endosomes to restore the pH. This accumulation of protons in the vesicle must be balanced by an influx of counter ions, which, in turn, ultimately causes osmotic swelling and destabilization of the endosomal membrane [19], thereby facilitating the endosomal escape of material trapped within the endosome.

In the current study, we compared the efficacy of DNA vectors fabricated from methylglycol-chitosan (MG-CS) and diethylaminoethyl-chitosan (DEAE-CS). MG-CS is a commercially available, water-soluble, cationic polymer that bears quaternized [–NMe_3_]^+^ and O(C_2_H_4_O)_n_H groups at the C-2 and C-6 atoms, respectively, of the pyranose ring. The MG-CS derivative can efficiently bind DNA to form self-assembling polyplexes via electrostatic interactions. However, the study of this chitosan derivative as a gene carrier has never been reported. The second water-soluble, but uncharged, chitosan derivative, DEAE-CS, is characterized by an increased buffer capacity because it bears tertiary amino moieties –CH_2_CH_2_N(CH_2_CH_3_)_2_. One study that investigated DEAE-CS as a non-viral DNA delivery carrier showed that it exhibited a comparable transfection efficiency in HeLa cells to that of the commercial lipid-based transfection agent Lipofectamine [20].

The abundance of quaternized positively charged [–NMe_3_]^+^ moieties in the MG-CS backbone allows it to interact with polyanionic DNA efficiently, thereby protecting bound DNA from enzymatic degradation by nucleases. However, MG-CS is not a proton-sponge polymer; thus, it is expected to show only limited ability to escape the endosome. Conversely, the DEAE-CS interacts with DNA to a lesser degree, but its increased buffer capacity should enhance the endosomal escape of the polyplexes by the proton-sponge mechanism. Therefore, although both vectors share a similar chemical structure, each has only one specific advantage: either a high concentration of positively charged moieties in its backbone (MG-CS) or a high buffer capacity (DEAE-CS). Transfection studies were carried out on epithelial cells and were extended to include dendritic cells to explore the potential application of these polymers as DNA vaccines.

## 2. Materials and Methods

### 2.1. Materials and Reagents

Crab chitosan with a degree of acetylation (DA) of 0.16 ± 0.02 (determined by ^1^H NMR and elemental analysis) and *M*_w_ of (8.14 ± 0.16) × 10^4^ g/mol (determined by size exclusion chromatography coupled to multi-angle light scattering (SEC-MALS)) was purchased from CJSC Bioprogress (Moscow, Russia). 2-Chloro-*N*,*N*-diethylethylamine hydrochloride (DEAE-Cl) and *O*-hydroxyethyl-*N*,*N*,*N*-trimethylchitosan iodide (methylglycol-chitosan, MG-CS) were purchased from Sigma Aldrich (Saint Louis, MO, USA). Dialysis membrane (MWCO 12,000–14,000) was obtained from Orange Scientific (Braine-l’Alleud, Belgium).

UltraPure™ agarose gel powder was purchased from Invitrogen (Carlsbad, CA, USA). GelRed™ nucleic acid stain (10,000× in water) was obtained from Biotium (Hayward, CA, USA). Gel loading solution was purchased from Sigma Aldrich and was mixed at a 1:6 ratio with sample before loading. The 10× UltraPure™ TAE Buffer, consisting of 400 mM Tris-acetate and 10 mM EDTA (pH 8.5, Invitrogen), was diluted with distilled water to prepare 1× Tris-acetate-EDTA (TAE) electrophoresis running buffer.

Plasmid DNA (gWiz™ Luciferase, 6732 bp) was purchased from Aldevron (Fargo, ND, USA). Lipofectamine™ 2000 (henceforth ‘Lipofectamine’) was obtained from Invitrogen and used as the positive control. The luciferase assay system was purchased from Promega (Madison, WI, USA). The Bio-Rad Protein Assay Dye Reagent Concentrate (5×) was purchased from Bio-Rad Laboratories (Hercules, CA, USA). Bovine serum albumin (BSA) was obtained from Sigma Aldrich.

Other chemicals, if not otherwise stated, were obtained from commercial sources and were used without purification. All solvents were of reagent grade and used as received.

### 2.2. Synthesis of DEAE-CS_I_ under Heterogeneous Conditions

Chitosan (1.0 g) was mixed with 2 mL of distilled water and 1.5 mL of 35% NaOH solution, and the mixture was stirred for 2 h using a magnetic stirrer. 2-chloro-*N*,*N*-diethylethylamine hydrochloride (5.35 g) was dissolved in 21.5 mL of 85% propan-2-ol (IPA: isopropyl alcohol) and added to the alkaline suspension of chitosan. This reaction mixture was stirred for 3 h at 80 °C under a nitrogen flow and then neutralized with 10% HCl. The DEAE-CS_I_ product was purified by dialysis against distilled water, tested for an absence of chloride ions, and freeze-dried.

### 2.3. Synthesis of DEAE-CS_II_ under Homogeneous Conditions

Chitosan (1.0 g) was dissolved in 10 mL of 0.1 M HCl. A solution of 2-chloro-*N*,*N*-dimethylethylamine (5.35 g in 15 mL of distilled water) was then added to the acidic polymer solution, and the pH was adjusted to 6.5 with sodium acetate. The reaction mixture was stirred at 80 °C for 3 h, followed by addition of 5 mL of 10% HCl. The product was dialyzed against distilled water, tested for an absence of chloride ions, and freeze-dried.

### 2.4. Characterization Methods

^1^H NMR spectra were measured on Bruker Avance II 400 MHz spectrometers (Bruker, Billerica, MA, USA). Samples were prepared by dissolving 5 mg of polymer in D_2_O/CF_3_COOH or D_2_O/DCl. Spectra were acquired at 323 or 343 K with suppression of the DOH signal. Integral of the H-1 signals of the glucosamine moieties of chitosan and its derivatives was used as a reference.

Elemental analysis was performed using a Perkin-Elmer Elemental Analyzer (Perkin-Elmer, Waltham, MA, USA).

### 2.5. Size Exclusion Chromatography Coupled to Multi-Angle Light Scattering (SEC-MALS)

Analytical fractionation was performed at room temperature on a system consisting of a PL Aquagel guard column (Polymer Labs, Amherst, MA, USA), followed by in-series PL Aquagel-OH 60, PL Aquagel-OH 50, and PL Aquagel-OH 40 analytical columns. The samples (100 µL injection volume; ca. 3.0 mg/mL in acetate buffer) were eluted with acetate buffer (pH 5, 0.2 M) at a flow rate of 0.7 mL/min (LC-20AD, Shimadzu, Milton Keynes, UK). The eluent was detected online [21] using multi-angle light scattering (MALS) (Dawn EOS, Wyatt Technology, Santa Barbara, CA, USA) and differential refractive index (Optilab rEX, Wyatt Technology, Santa Barbara, CA, USA) detectors. The absolute weight-average molar masses (*M*_w_) and polydispersity indices ($\frac{M_{w}}{M_{n}}$) were calculated using the ASTRA^®^ (Version 5) software (Wyatt Technology, Santa Barbara, CA, USA) and the refractive index increment, *dn/dc* = 0.163 mL/g for chitosan and chitosan derivatives [22,23,24].

### 2.6. Dynamic Light Scattering (DLS)

A solution of DNA in Dulbecco’s Modified Eagle Medium (DMEM) was mixed with a volume of chitosan solution determined based on the polysaccharide: DNA mass ratio and then diluted up to 1 mL with DMEM medium to obtain a final DNA concentration of 6.29 µg/mL. The solutions were vortexed and left to stand for 10 min to allow completion of the complex formation. The sizes of the complexes were determined with a Zetasizer Nano S90 instrument (Malvern Panalytical, Almelo, The Netherlands) (scattering angle of 173° at 25 °C). The average complex size was calculated as a mean value of three measurements.

### 2.7. Gel-Retardation Assay

Gel electrophoresis was performed in 1% agarose gel in TAE buffer at a voltage of 120 V using a Bio-Rad Sub-cell GT Basic setup. The intercalating agent was 3,8-diamino-5-ethyl-6-phenylphenantridium bromide. DNA was detected with a BioRad Molecular Imager ChemiDoc XRS system (BioRad Laboratories, Hercules, CA, USA).

### 2.8. Ethidium Bromide Displacement Assay

An ethidium bromide (EtBr) displacement assay was performed according to Oliveira et al. [20], with minor modifications. Briefly, 2.5 µL of EtBr solution in water (2.5 × 10^−3^ M) was added to 2 mL of phosphate buffer saline (PBS, pH 7.4). The DNA stock solution was then added to final DNA concentration of 5 ng/µL. The solution was then titrated with polysaccharide solution in PBS (1 mg/mL) from mass ratios of 0.1:1 to 10:1. Measurements were recorded using a Shimadzu RF-5301PC spectrofluorometer (λ_ex_ = 560 nm; λ_em_ = 605 nm).

### 2.9. Cell Culture

Calu-3 cells (human lung adenocarcinomic bronchial epithelial cells) and JAWSII cells (mouse immature dendritic cells) were obtained from American Type Culture Collection (Manassas, VA, USA). Calu-3 cells were grown in DMEM/F-12 medium supplemented with 10% fetal bovine serum (FBS) and were cultured once a week; JAWSII cells were cultured in α-minimum essential medium supplemented with 20% FBS and 5 ng/ml recombinant mouse granulocyte-macrophage colony stimulating factor (GM-CSF) and were subcultured once weekly. All the cell culture media contained 1% antibiotic/antimycotic liquid, and all cells were maintained at 37 °C in a 5% CO_2_ atmosphere.

### 2.10. DNA Transfection

Calu-3 cells and JAWSII cells in 24-well plates were transfected with chitosan derivative/DNA complexes by adding 1 μg luciferase plasmid per well. Lipofectamine was used as a control. The complexes were prepared at different chitosan to DNA mass ratios in Opti-MEM I reduced serum medium, with a final volume of 400 μL per well. The cells were transfected when they had reached 70–80% confluency. After incubating the cells with the complexes for 5 h at 37 °C, the transfection medium was removed and replaced with serum-supplemented cell culture medium. At 24 h post-transfection, the medium was aspirated, and the cells were lysed in 100 μL Promega reporter lysis buffer. The plates were then subjected to three freeze-thaw cycles to achieve complete cell lysis prior to the luminescence detection step. The luciferase expression was detected using the luciferase assay system according to the manufacturer’s protocol. In brief, 20 μL of cell lysate was transferred to a black 96-well plate, and the relative light unit (RLU) was detected by a luminometer (MLX Microtiter^®^ Plate Luminometer, Dynatech Laboratories, Inc. Chantilly, VA, USA) with an auto-feeding and detection system. The luminometer was programmed to inject 100 μL of reconstituted luciferase assay substrate per well and to perform a 2 s luminescence measurement (RLU). The results were expressed as relative light units (RLU) per mg of total protein.

### 2.11. Bradford Protein Quantification

After transfection, the cell lysates were centrifuged at 12,000× *g* for 2 min at 4 °C. The concentration of total protein in the cell supernatant was determined by the Bradford method. The Bradford protein assay reagent (5×) was diluted to a 1× working solution with distilled water and filtered through filter paper. Bovine serum albumin (BSA), dissolved in distilled water at 1 mg/mL, was used to prepare a protein standard curve with concentrations ranging from 0.2 to 1 mg/mL. Sample solutions and protein standards were added to a 96-well plate and mixed with 200 μL 1× Bradford protein assay reagent for 5 min on a plate shaker at room temperature. The absorbance of the samples was measured at 595 nm with an Epoch 96-well Microplate Spectrophotometer (BioTek Instruments Inc., Winooski, VT, USA). Sample protein concentrations were calculated from the BSA standard curve.

### 2.12. Assessing the Toxicity In Vitro

The toxicity of the polymers in Madin-Darby canine kidney (MDCK) cells was assessed by serially diluting the polymer stock solutions in alpha-MEM maintenance medium. These solutions, in 6 replicates, were added in amounts of 0.1 mL per well to 96-well plates containing confluent MDCK cell monolayers (~10,000 cells). After incubation for 24 h at 37 °C in a 5% CO_2_ atmosphere, a micro tetrazolium assay was performed. The cells were washed twice with phosphate buffered saline (PBS), and then 0.1 mL of 0.5 µg/mL 3-(4,5-dimethylthiazol)-2,5-diphenyltetrazolium bromide (MTT) solution in PBS was added to each well. After a 1 h incubation (37 °C and 5% CO_2_), the medium was removed and 0.1 mL of 95% C_2_H_5_OH was added to each well to dissolve the (*E*,*Z*)-5-(4,5-dimethylthiazol-2)-1,3-diphenyformazan (formazan) crystals. The optical density was measured at 535 nm using a multifunctional CLARIOstar^®^ reader (BMG LABTECH, Ortenberg, Germany). The obtained results were used to calculate the LC_50_ of the chitosan derivatives. The toxicity of Lipofectamine 2000 was assessed under the same conditions at amounts of 1.25–5 µL per well, as recommended by the manufacturer’s protocol.

### 2.13. Statistical Analysis

The data were analyzed using one-way ANOVA followed by a Bonferroni *post-hoc* test. The analysis was performed for each derivative independently with *q* = 3 using naked DNA and Lipofectamine as controls in transfection and cytotoxicity experiments, respectively. Calculations were performed using online service astatsa.com.

## 3. Results and Discussion

### 3.1. Characterization of MG-CS

**Figure 1:**
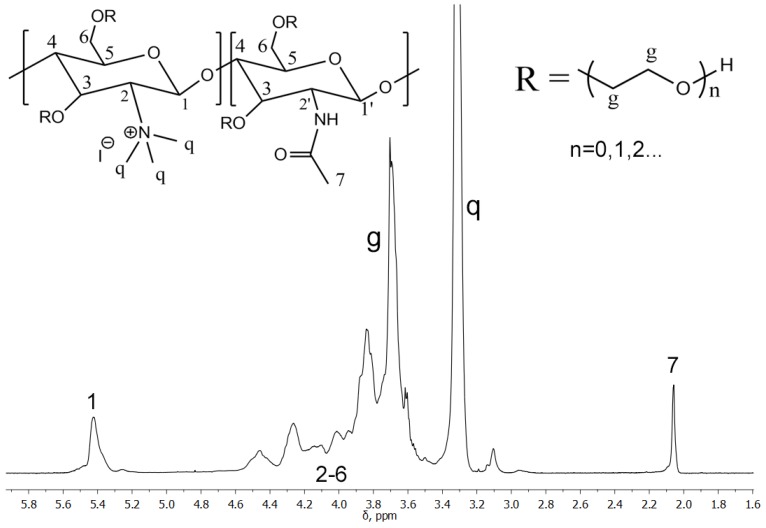
^1^H NMR spectrum of methylglycol-chitosan (MG-CS) (323 К, 400 MHz, D_2_O + DCl).

Previous studies unambiguously demonstrated a strong dependence of the key characteristics of chitosan-based gene therapy vectors, such as their transfection efficiency and cytotoxicity, on the *M*_w_, the degree of acetylation (DA), and the degree of substitution (DS) of the chitosan derivative used [11]. MG-CS (Figure 1) possesses hydroxyethyl moieties at C-6 and C-3 atoms and quaternized [–N(CH_3_)_3_]^+^ moieties at the C-2 atom of the pyranose ring. Thus, the *M*_w_ and DS (denoted, in the case of MG-CS, as the degree of glycolization, DG), the DA, and the degree of quaternization (DQ) are important structural characteristics that influence the transfection and cytotoxic properties of MG-CS-based vectors.

The MG-CS used here was obtained from a commercial source and was characterized by ^1^H NMR spectroscopy to evaluate its DA, DG and DQ. The NMR spectrum of MG-CS (Figure 1) displayed all the characteristic signals of the chitosan backbone: 3.5–4.6 (H-2,3,4,5,6), 5.43 (H-1 from GlcN^+^Me_3_). The signals of *N*-methyl protons were represented by a sharp singlet at 3.34 ppm.

The DA of MG-CS was calculated by integration of the acetamide signal according to the following equation:

(1) $$\mathrm{DA}=\frac{I_{\left(7\right)}}{3+I_{\left(7\right)}}$$

The glycol moiety signals were overlapped by polysaccharide backbone signals, so the DG was calculated as the increase in integral intensity in the region from 3.5–4.6 ppm, according to the following equation:

(2) $$\mathrm{DG}=\frac{3\left(I_{\left(2-6,g\right)}-2I_{\left(7\right)}-6\right)}{12+4I_{\left(7\right)}}$$

The DQ, calculated using the following formula,

(3) $$\mathrm{DQ}=\frac{I_{\left(q\right)}}{9+3I_{\left(7\right)}}=0.87$$

indicates a complete quaternization of the chitosan amino groups. All integrals were normalized with respect to the H-1 signals of the glucosamine moieties of MG-CS. The results are shown in Table 1.

### 3.2. Synthesis and Characterization of DEAE-CS

DEAE-CS was synthesized using two different protocols involving the interaction of DEAE-Cl and chitosan. The first protocol (Section 2.2) included the reaction between chitosan and DEAE-Cl under heterogeneous conditions in an alkaline (IPA-NaOH) medium. The second procedure (Section 2.3) involved treatment of chitosan with DEAE-Cl in acetate buffer solution under homogeneous conditions at pH 6.5. In both cases, we obtained water-soluble chitosan derivatives (DEAE-CS_I_ and DEAE-CS_II_, respectively), which were then characterized by ^1^Н NMR spectroscopy.

**Figure 2:**
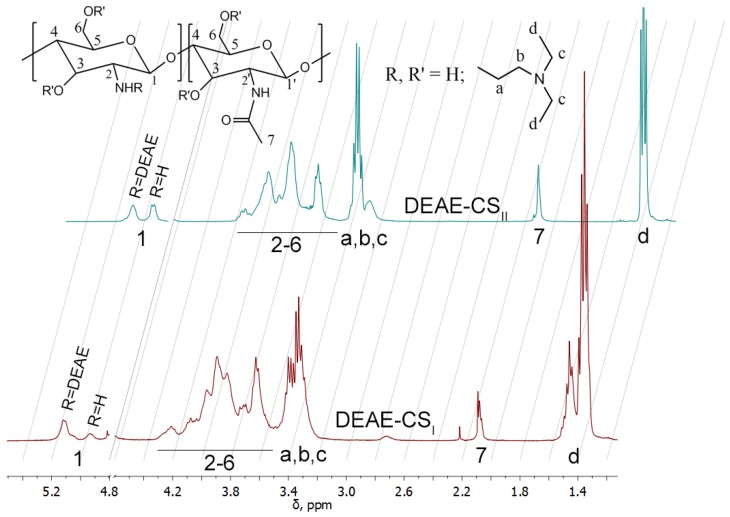
^1^Н NMR spectra of diethylaminoethyl-chitosan (DEAE-CS_I_ and DEAE-CS_II_ with degrees of substitution of 1.2 and 0.57, respectively) (343 К, 400 MHz, D_2_O + CF_3_COOH).

The ^1^H NMR spectrum of DEAE-CS_II_ (Figure 2) shows three signals that provided information about the degrees of substitution. The triplet at 1.35 ppm corresponds to the methyl group protons of the CH_3_–CH_2_– moiety. *N*-substitution is confirmed by a shift in the anomeric proton signal to the weak field (5.1 ppm). The methylene protons of the CH_3_–CH_2_– moiety are represented by a strong signal at 3.33 ppm. These signals are overlapped with the H-2 signal of the glucosamine units. The DS of DEAE-CS was calculated according to the following equation:

(4) $$\mathrm{DS}=\frac{I_{\left(d\right)}}{6+2I_{\left(7\right)}}$$

The ^1^H NMR spectrum of the DEAE-CS_I_ synthesized in IPA-NaOH medium (Figure 2) indicates the generation of highly substituted products. Thus, treatment of chitosan with a fivefold excess of DEAE-Cl in IPA-NaOH generated DEAE-CS with DS = 1.2, while treatment in acetate buffer solution at pH 5–6 for the same duration and with the same concentration of reactants and the same temperature gave a product with DS = 0.57. The anomeric proton shift in DEAE-CS_I_ shows that about 75% of the amino groups are substituted. Methyl protons of the DEAE substituent are represented by three superimposed triplets that can be explained by the presence of *O*- and *N*-substitution and alkylation of the tertiary amino group by DEAE-Cl. However, further investigations are required for determination of the molar fractions of each substituent.

In the case of DEAE-CS_II_, only one kind of substituent is observed, and this is also confirmed by the correlation between the integrals of the methyl group and the shifted anomeric proton. Integration of the acetamide proton (2.08 ppm) shows no occurrence of deacetylation in either the acetate buffer solution or the IPA-NaOH medium (Table 1).

Thus, DEAE-CS with different DS was synthesized by a convenient and straightforward one-stage protocol. The reaction between chitosan and DEAE-Cl under homogeneous conditions in acetate buffer solution leads to the formation of an *N*-substituted DEAE-CS_II_ with a moderate DS, whereas the reaction conducted under heterogeneous conditions in IPA-NaOH medium gives rise to a highly substituted DEAE-CS_I_.

The SEC-MALS data (Table 1) showed that all the polymers used in our study had comparable *M*_w_ (around 10^5^ g/mol). The *M*_w_ of DEAE-CS increased with increases in the DS. The commercial sample of MG-CS had the lowest polydispersity index (PDI), which was almost half that of chitosan. By contrast, the synthesis of DEAE-CS decreased the PDI, which may be explained by diffusion of the low molecular weight fractions through the dialysis membrane.

### 3.3. Assessing the Toxicity In Vitro

**Figure 3:**
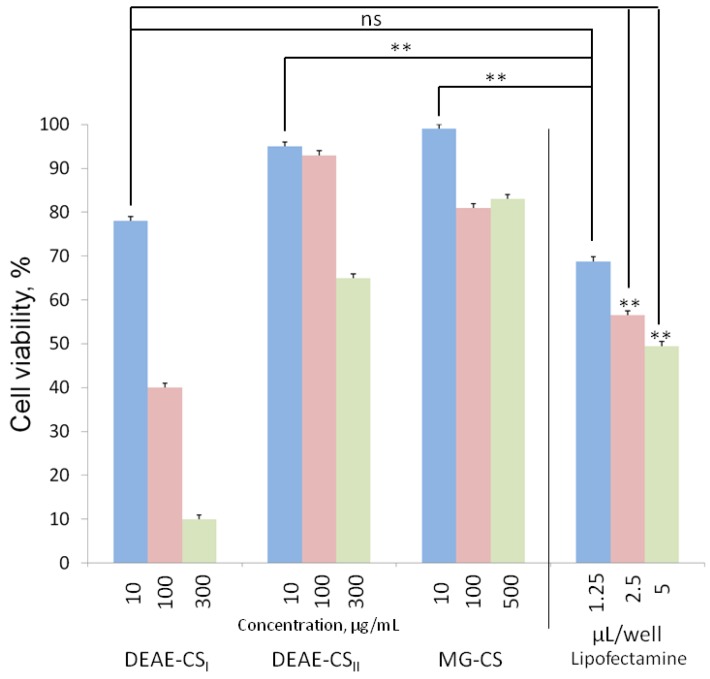
Cell viability after 24 h of incubation with chitosan derivatives. Values are the mean ± standard deviation; measurements were performed as 3 individual experiments with 6 technical replicates. Calculated LC_50_(DEAE-CS_I_) = 80 µg/mL, LC_50_(DEAE-CS_II_) = 500 µg/mL. ** *p* < 0.01; ns *p* > 0.05.

The MTT assay showed that all the studied chitosan derivatives exhibited concentration-dependent cytotoxicity. MG-CS had the lowest toxicity since it decreased cell viability only to 80% even at the highest concentration studied. The cytotoxicity of DEAE-CS was determined by its DS. Increasing the DS from 0.57 to 1.2 diminished cell viability from 65% (for DEAE-CS_II_) to 10% (for DEAE-CS_I_) (Figure 3). Note that the highest polysaccharide concentration used in transfection experiments was 2.5 µg/mL (at 10:1 ratio). Four times higher polysaccharide concentration (10 µg/mL) was less harmful in most cases than the lowest studied Lipofectamine dose (Figure 3). Therefore, all three chitosan derivatives proved to be significantly less cytotoxic than Lipofectamine. Hence they are considered to be safer and more suitable than Lipofectamine for therapeutic use.

### 3.4. Evaluation of DNA Binding Affinity

**Figure 4:**
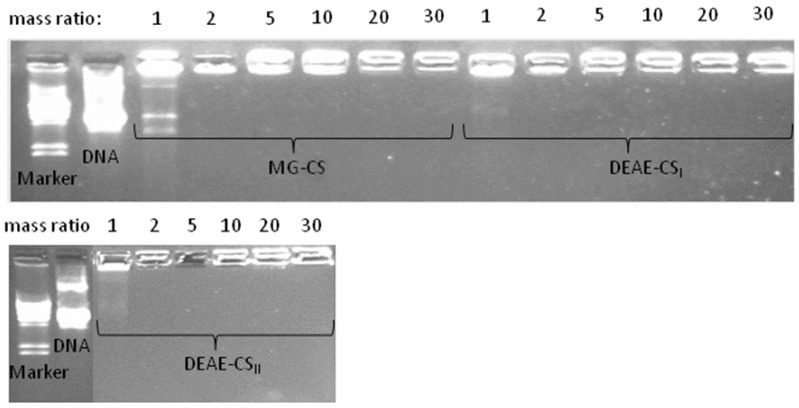
Gel retardation analysis of polysaccharide: DNA mixtures at mass ratios of 1:1, 2:1, 5:1, 10:1, 20:1, and 30:1.

The DNA binding ability of DEAE-CS and MG-CS was studied by agarose gel electrophoresis. Polyplexes of MG-CS, DEAE-CS_I_, and DEAE-CS_II_ were prepared at different polysaccharide:DNA mass ratios (1:1, 2:1, 5:1, 10:1, 20:1 and 30:1). Figure 4 demonstrates that the DNA migration was completely retarded at a polysaccharide:DNA mass ratio of 2:1. This can be explained by complete DNA binding and negative charge neutralization.

We quantitatively evaluated the process of DNA binding by chitosan derivatives with a fluorescent intercalator (EtBr) displacement assay. This method is based on the competitive displacement of a cationic dye by cationic polysaccharides, which results in fluorescence quenching.

**Figure 5:**
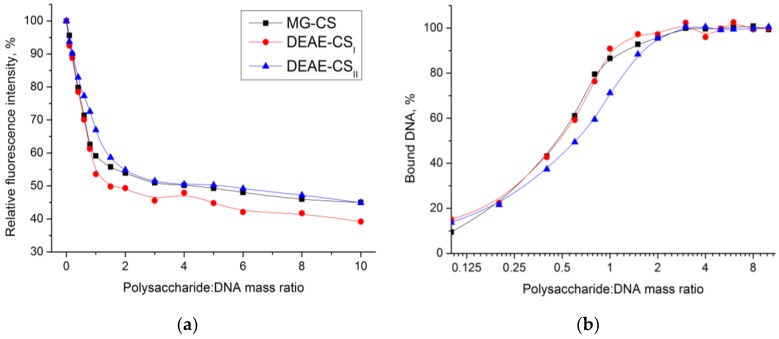
Ethidium bromide displacement curves represented as (**a**) Relative fluorescence intensity quenching; (**b**) Bound DNA fraction as a function of polysaccharide: DNA mass ratio; *n* = 3.

As shown in Figure 5a, fluorescence quenching curves can be described by two linear fragments and a smooth transition between them. At low polysaccharide:DNA mass ratios, the fluorescence intensity rapidly decreases due to polyplex formation. Around the stoichiometric ratio, a smooth transition represents a dynamic equilibrium of the polyplex association-dissociation processes, whereas an excessive polysaccharide amount results in a slow linear decrease due to nonspecific fluorescence quenching.

The relative fluorescence intensity (*Q*, %) and the fraction of bound DNA (*B*, %) were calculated using the following formulas:

(5) $$Q=\frac{F_{x}-F_{EB}}{F_{0}-F_{EB}}\times 100,\;B=\frac{100-Q}{100-\left(aR+b\right)}\times 100,$$

where *F_x_*, *F*_0_, and *F*_EB_ are the fluorescence intensities at *x* equivalents of polysaccharide added, before titration, and for the blank EtBr solution, respectively; *R* is the polysaccharide:DNA mass ratio; *a* and *b* are empirical constants of post-saturation curve fragment linear fitting.

The intersection of the linear curve fragments provides information about the stoichiometric polyplex composition. This was 1.1:1 for both MG-CS and DEAE-CS_I_ 1.2:1 for DEAE-CS_II_. As shown in Figure 5b, at a mass ratio higher than 2:1, more than 95% of the DNA is bound in polyplexes. This fact corresponds well with the gel retardation assay data.

### 3.5. DLS Measurements

Several previous studies have shown that the penetration of polyplexes through cell membranes strongly depends on their size. The optimal size for transfection is generally accepted as about 200 nm or less [25,26,27]. In the present study, the dependence of polyplex size on polymer composition was studied using DLS.

**Figure 6:**
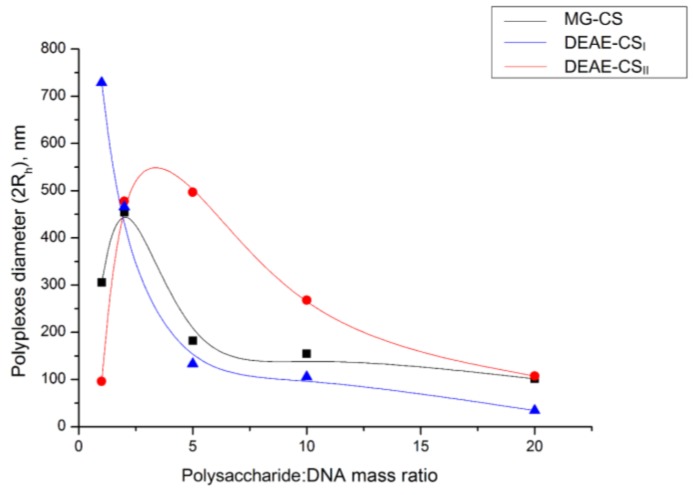
Hydrodynamic diameters of polyplexes formed by chitosan derivatives and DNA at different mass ratios.

Polyplexes based on three derivatives (DEAE-CS_I_, DEAE-CS_II_, and MG-CS) were prepared at five different polysaccharide:DNA mass ratios of 1:1, 2:1, 5:1, 10:1, and 20:1. All polyplexes revealed similar patterns of hydrodynamic diameter (2*R*_h_), which changed at different ratios. At some ratios, the size increase ceases and is followed by a gradual decline (Figure 6). The effect of these size decreases can be explained by a more effective nucleic acid binding and by electrostatic repulsive forces that prevent aggregation among the complexes.

### 3.6. In Vitro DNA Transfection Efficiency

**Figure 7:**
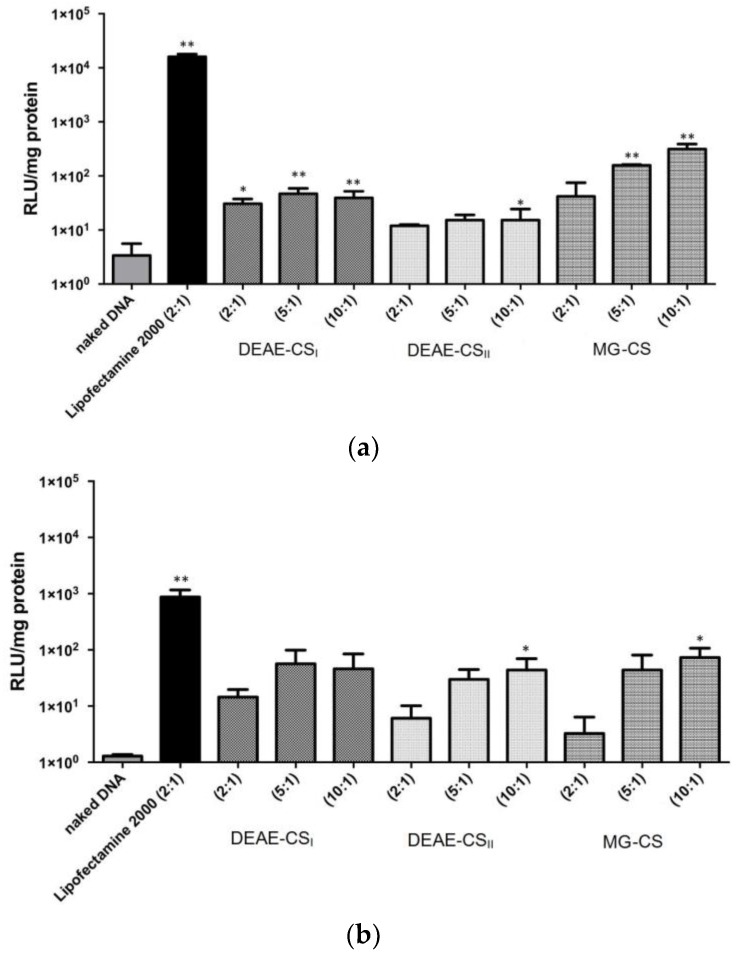
Transfection of Calu-3 (**a**) and JAWSII (**b**) cell lines. Values are the mean ± standard deviation; *n* = 3. Statistical analysis is presented in Table A1; * *p* < 0.05; ** *p* < 0.01.

The in vitro transfection was performed in Calu-3 and JAWSII cells. Polyplexes based on DEAE-CS_I_, DEAE-CS_II_, and MG-CS were formulated at different polysaccharide:DNA ratios (2:1, 5:1, and 10:1) and compared to Lipofectamine:DNA complexes (ratio 2:1). The transfection efficiency in Calu-3 cells was lower for both the DEAE-CS and MG-CS derivatives than for Lipofectamine. The highest transfection efficiencies were obtained from chitosan-based particles with a 10:1 mass ratio, which is in line with our DLS data showing that, at the 10:1 ratio, all derivatives form complexes with sizes close to 200 nm (Figure 7a). In general, higher transfection efficiency was achieved when the polyplexes were prepared at higher polysaccharide:DNA ratios, possibly due to the formation of smaller polyplexes that facilitated cellular uptake and presence of free cationic chains promoting the endosomal escape.

As expected, a slightly better efficiency was achieved in transfection experiments with DEAE-CS_I_ (with a higher DS) than with DEAE-CS_II_. Moreover, within the same DS, the transfection efficiency of DEAE-CS derivatives was poorly correlated with the mass ratio. However, in the case of MG-CS:DNA complexes, the mass ratio had a marked effect on transfection, as the effectiveness of MG-CS improved with an increase in the polysaccharide:DNA ratio. Figure 7a shows that the difference in the transfection efficiency of DEAE-CS_I_ and MG-CS at a polysaccharide:DNA ratio = 2:1 was not statistically significant.

In general, transfection was poorer in JAWSII cells than in Calu-3, HeLa, and many other cell lines [28,29]. In this context, a comparison of the transfection activity of DEAE-CS and MG-CS vectors versus Lipofectamine into JAWSII cells was of interest. The results of these experiments are presented in Figure 7b. The transfection efficiency of DEAE-CS_I_, DEAE-CS_II_, and MG-CS at polysaccharide:DNA ratios of 5:1 and 10:1 showed no statistically significant difference. However, the expression level of luciferase was higher for DEAE-CS_I_ at a polysaccharide:DNA ratio of 2:1 than for DEAE-CS_II_ and MG-CS polyplexes formulated at the same ratio. In general, the efficacy of Lipofectamine and MG-CS decreased in experiments with JAWSII cells when compared to experiments conducted using the Calu-3 cells, whereas the efficiency of DEAE-CS remained close to that determined in the previous Calu-3 experiment.

## 4. Conclusions

The following conclusions can be drawn from the results of this work:

1. The interaction of DEAE-Cl with chitosan at pH 5–6 furnishes *N*-substituted DEAE-CS_II_ with a moderate DS, whereas the reaction in alkaline medium leads to a highly substituted product with a more uncertain pattern of substitution.
2. Both DEAE-CS and MG-CS efficiently bind DNA to form stable self-assembled polyplexes at polysaccharide:DNA ratios greater than 2:1. The stoichiometry of the polyplexes was about 1:1.
3. In Calu-3 cells, the DNA transfection activity of MG-CS improves with increasing polysaccharide:DNA ratios. By contrast, the transfection efficiency of DEAE-CS is lower and has no expressed dependency on the DS or the polysaccharide:DNA ratio. In JAWSII cells, both Lipofectamine and MG-CS show significantly less DNA transfection activity, while the efficiency of DEAE-CS remains at the same level as seen in Calu-3 experiments (or even slightly increases).
4. The toxicity of DEAE-CS rises with increasing concentrations of the polymer and its DS, whereas MG-CS demonstrates only moderate cell toxicity, even at the highest concentration studied.

Overall, MG-CS proved to be a more efficient and significantly less toxic vector when compared to DEAE-CS. However, DEAE-CS is also of interest for two reasons: it does not lose its effectiveness in the transfection-recalcitrant JAWSII line, and the synthesis of this polymer is a one-stage and straightforward reaction. By contrast, MG-CS must be synthesized in several stages, including protection-deprotection, and some of these steps proceed under harsh conditions. Taken together, the results from in vitro experiments confirmed that both DEAE-CS and MG-CS are promising natural-based gene delivery vectors that need further study, especially in in vivo conditions.

## Figures and Tables

**Table 1:** Characteristics of the chitosan derivatives.

Sample	DS ^1^	DA ^1^	*M*_w_^2^ (g/mol)	PDI = *M*_w_/*M*_n_^2^	Elemental Composition (%)		
					C	H	N
MG-CS	1.5 (DG)	0.13	114,000 ± 3000	1.36 ± 0.07	30.8	4.95	2.91
	0.87 (DQ)						
Chitosan	-	0.15	81,400 ± 1600	2.10 ± 0.40	36.6	8.21	6.54
		(0.17) ^3^					
DEAE-CS_I_	1.2	0.17	134,000 ± 1000	1.68 ± 0.08	45.1	7.61	7.51
DEAE-CS_II_	0.57	0.18	84,500 ± 1700	1.64 ± 0.07	39.8	8.08	7.13

^1^ Determined by ^1^H NMR spectroscopy; ^2^ Determined by SEC-MALS; ^3^ Determined from elemental analysis data.

## Appendix A

**Table A1:** Statistical analysis of the transfection experiments.

Pair vs. Naked DNA		Calu-3 Cells		JAWSII Cells	
		Bonferroni *p*-Value	Significance	Bonferroni *p*-Value	Significance
MG-CS	2:1 ^1^	0.85	ns	2.8	ns
	5:1	0.005	**	0.22	ns
	10:1	5 × 10^−5^	**	0.026	*
DEAE-CS_I_	2:1	0.025	*	1.8	ns
	5:1	0.002	**	0.14	ns
	10:1	0.005	**	0.28	ns
DEAE-CS_II_	2:1	0.21	ns	2.1	ns
	5:1	0.061	ns	0.14	ns
	10:1	0.025	*	0.025	*

^1^ Polysaccharide: DNA mass ratio; * *p* < 0.05; ** *p* < 0.01; ns *p* > 0.05.
